# The impact of COVID-19 on people ageing with an intellectual disability in Ireland: Protocol for a follow-up survey

**DOI:** 10.12688/hrbopenres.13340.1

**Published:** 2021-08-26

**Authors:** Mary McCarron, Andrew Allen, Darren McCausland, Margaret Haigh, Retha Luus, Fathima Rosmin Bavussantakath, Fintan Sheerin, Niamh Mulryan, Eilish Burke, Eimear McGlinchey, Fidelma Flannery, Philip McCallion

**Affiliations:** 1 Trinity Centre for Ageing and Intellectual Disability, Trinity College Dublin, Dublin 2, Ireland; 2 School of Social Work, Temple University, Philadelphia, Pennsylvania, PA 19122, USA

**Keywords:** COVID-19, frailty, healthcare, intellectual disability, mental health, lockdown, social participation, vaccination

## Abstract

**Background**:  The COVID-19 pandemic and associated lockdowns have had a dramatic impact on many people, but individuals with an intellectual disability, given the prevalence of congregate living and high levels of co-morbid conditions, may be particularly vulnerable at this time. A prior initial survey of participants of the Intellectual Disability Supplement to the Irish Longitudinal Study on Ageing (IDS-TILDA) found that, despite a majority of participants being tested, only a small proportion had tested positive for COVID-19. Furthermore, despite some reporting positive aspects to the lockdown, a similar proportion were experiencing stress or anxiety during the pandemic. The pandemic and lockdowns have continued, and it is possible that experiences and consequences have changed over time.

**Aim**: To explore over time and in greater depth the impact of COVID-19 and associated lockdowns and to further establish rates of infection, rates of vaccination and participants’ experiences.

**Methods**: A structured questionnaire for people with intellectual disability participating in the IDS-TILDA longitudinal study, to be administered by telephone/video in summer 2021. Where participants are unable to respond independently, a proxy respondent will be invited to either assist the participant or answer questions on their behalf. This questionnaire will include questions from the first COVID-19 questionnaire, with extra questions assessing “long COVID” (i.e. COVID-19 lasting for 12 weeks or longer), infection control behaviours, changes in mental health, social contacts and loneliness, frailty, healthcare, and incidence of vaccination.

**Impact**: The results of this survey will be used to inform healthcare provision for people with intellectual disability during the latter stages of the lockdown and into the future.

## Introduction

The COVID-19 pandemic has had an unprecedented effect on society at large, and our understanding of this pandemic is constantly changing as research continues at a rapid pace. Besides the economic effect of ongoing lockdowns, the global death toll as of early June 2021 is estimated to be approximately 4.2 million ( WHO, 2021). Although the impact of this pandemic has been devastating for many, it has been particularly severe for the more vulnerable members of society. People with an intellectual disability are an especially vulnerable group ( Courtenay & Perera, 2020), and evidence from the UK and Ireland found that deaths attributed to COVID-19 occurred at a younger age for people with intellectual disability compared to the general population ( Perera *et al.*, 2020). Data from an international study (with participants predominantly from the Unites States) indicated that the case-fatality rate was similar for people with an intellectual disability overall, but was higher for those age 18–74 ( Turk *et al.*, 2020). Apart from the threat of serious illness or death, measures employed to reduce the spread of COVID-19 have had an additional major effect on the lives of people with intellectual disability. People with an intellectual disability have missed social contacts, have had their daily lives altered by being unable to leave their homes, and have often found it hard to understand the preventative measures ( Embregts *et al.*, 2020). Survey respondents in Spain reported differing conditions of support for people with intellectual disability during the COVID-19 pandemic depending on living context; for example, those in family homes relied heavily on their family for support ( Navas *et al.*, 2021).

The disproportionate effect of this pandemic has led to relevant stakeholders advocating for priority for vaccinations to be given to people with intellectual disability ( Hotez *et al.*, 2021). Notwithstanding the ongoing rollout of vaccinations, at time of writing lockdown restrictions continue in Ireland, and the pandemic may have effects that persist well beyond the end of any societal restrictions. Given existing challenges in planning for long-term care for many adults with intellectual disability, the COVID-19 pandemic has further added to the vulnerability of this cohort.

The Intellectual Disability Supplement to the Irish Longitudinal Study on Ageing (IDS-TILDA) was established in 2008 to study ageing in a representative sample of people with intellectual disabilities in Ireland aged 40 and older. With the onset of the COVID-19 pandemic and the associated lockdown in mid-March 2020, all data collection for Wave 4 of IDS-TILDA, which was ongoing at that time, was suspended. However, ethical approval was obtained in May 2020 to complete Wave 4 data collection by remote interviewing (by phone/video) rather than face-to-face and for the addition of a supplemental COVID-19 survey to investigate the impact of the pandemic and lockdown on the IDS-TILDA participants.

This initial IDS-TILDA COVID-19 survey, conducted between May and September 2020, found no deaths from COVID-19 among participants ( McCarron *et al.*, 2020; McCarron *et al.*, 2021). Seven hundred and ten participants completed the COVID-19 questionnaire, out of 739 enrolled in Wave 4, a 96% response rate. Participants were aged over 40, with all levels of intellectual disability represented. A majority (62.4%) had been tested for COVID-19, although only 10% reported COVID-19-like symptoms and only 2.5% tested positive. These findings were despite high rates reported of chronic conditions associated with worse outcomes for COVID-19, including cardiovascular disease and diabetes. Of those participants who tested positive or had symptoms, over three-quarters had a plan in place to manage self-isolation according to guidelines, although one third were unable to comply with these guidelines. Most participants (58%) indicated there had been positive aspects to the lockdown (for example, trying new activities, more rest and relaxation), but 55% indicated they had experienced stress/anxiety, with most common reasons being not being able to do usual activities, not being able to see family/friends and fear of contracting COVID-19. The full report (including an accessible version) is available at: https://idstilda.tcd.ie/wave4/launch.php

There is a need for additional data collection. Ireland has had an extended, restrictive lockdown since January 2021, although a national vaccination program has been progressing, with over 1.4 million people in Ireland having received at last one vaccine dose at the time of writing. It is important to understand the impact of extended lockdown and the experience of vaccination for individuals ageing with an intellectual disability by repeating the questions from the first survey, and asking additional questions on mental health consequences, changes suggesting increased frailty and/or “long COVID”, and access to and experience of vaccination. The questions intended to be asked are an opportunity to gather data that is comparable to questionnaires being completed with other populations and in other countries. 

## Protocol

### Ethical approval

Ethics approval has been received from the National Research Ethics Committee (Application number: 20-NREC-COV-050-AMEND-2). 

### Participants

The National Intellectual Disability Database was originally used, with support from the Health Research Board, to anonymously recruit a representative sample of adults with an intellectual disability aged 40 years or older for IDS-TILDA. For Wave 4 the sample was refreshed from the same source ( McCarron *et al.*, 2020). The sample for this follow-up survey will again consist of participants in Wave 4. At time of writing, 725 participants from the original Wave 4 sample of 739 are surviving, and all will be invited to take part in the second COVID-19 survey. The questionnaire is openly available at the following link: https://idstilda.tcd.ie/data/datacollectiontools/

### Survey development

Existing questionnaires on the impact of the COVID-19 pandemic were consulted in the preparation of the current survey. The research team obtained feedback from the International Scientific Advisory Committee and drew on work with international collaborators including the T21RS (Trisomy 21 Research Society) group ( https://www.t21rs.org/covid-19/). The research team also consulted with the Steering Committee at the Trinity Centre for Ageing and Intellectual Disability, who provided feedback that was incorporated into the survey. As public and patient involvement (PPI) remains central to IDS-TILDA, a consultation session on the proposed questions was held with adults with intellectual disability. The adults, members of the Inclusive Research Network, provided perspectives of their lived experience of the pandemic which informed aspects of the protocol.

### Measures

The questionnaire (see Participants for link to the full questionnaire) will contain the following sections:

***1. Health and COVID-19***

Items gathered will include a rating of overall health (Likert scale, adapted from TILDA’s COVID-19 survey; Ward *et al.*, 2021); five items from the “physical health problems” section of the Epidemic-Pandemic Impacts Inventory (EPII; Grasso *et al.*, 2020); symptoms of COVID-19 and their duration; frequency of COVID-19 testing and results of tests (from first IDS-TILDA COVID-19 questionnaire; McCarron *et al.*, 2020, updated for more recently identified symptoms of COVID-19 and “long COVID”, i.e. COVID-19 lasting longer than 12 weeks); and contact with others who had COVID-19 (adapted from TILDA’s COVID-19 survey).

***2. Responding to COVID-19***

Questions will establish if the participant moved from their home as a result of COVID-19 concerns; was there a plan to manage self-isolation; did hospitalisation/admission to intensive care (first IDS-TILDA COVID-19 questionnaire), household quarantine, and/or limiting of physical closeness with loved ones occur (from “physical distancing and quarantine” section of EPII); and whether participant received accessible information and how easy it was to understand on a four-point Likert scale (new items).

***3. Infection control behaviours***

Personal responses to improve infection control will be assessed using five items (e.g. “did you wash your hands more frequently than usual”) (adapted from TILDA’s COVID-19 survey).

***4. Mental health during COVID-19 pandemic and lockdown***

As well as asking for a rating of overall mental health (Likert scale, adapted from TILDA’s COVID-19 survey), other items will address causes of stress/anxiety (first IDS-TILDA COVID-19 questionnaire); anxiety (GAD-7; Spitzer *et al.*, 2006, and Glasgow Anxiety Scale; Mindham & Espie, 2003); loneliness (UCLA loneliness scale; Russell *et al.*, 1980); depression (PHQ-9; Kroenke *et al.*, 2001); and whether the participant was able to access supports for mental health (new item). 

***5. Contact with others***

Established items will ask for information on contact with family/friends in person; in writing or via technology (adapted from IDS-TILDA Wave 4); reduced hours of work/day service (adapted from “work and employment” section of the EPII); arguments/physical conflict at home (adapted from “home life” section of the EPII); and social activities (“social activities” section of the EPII).

***6. Life events***

Data will be gathered on major life events and level of stress caused (none/a little/a lot; adapted from Hermans & Evenhis, 2012, and IDS-TILDA Wave 4 study); death of a person close to participant due to COVID-19 (Hatton, personal communication).

***7. Positive aspects of the COVID-19 period***

A series of questions will investigate positive aspects experienced during the COVID-19 period. (adapted from first IDS-TILDA COVID-19 questionnaire and EPII).

***8. Frailty***

Standardized questions will gather information on changes in strength; mobility; falls; weight; new diagnoses (adapted from SARC-F, Woo *et al.*, 2014; and FRAIL scale items, Morley *et al.*, 2012).

***9. Impact of COVID-19 on healthcare utilisation***

Addressing healthcare utilization questions will ask about appointment cancellations; whether health practitioners are usually seen once a year, and whether this occurred since pandemic (adapted from TILDA COVID-19 questionnaire); information on new healthcare appointments (based on consultation with Inclusive Research Network); changes in medication and health supplements (adapted from TILDA COVID-19 questionnaire).

***10. Vaccinations***

A new area for data gathering, questions will address whether easy read information was received and how easy it was to understand; whether vaccine was received; which vaccine; date(s) of dose(s); what it means to the individual to have received the vaccine; side effects reported and their duration; location where vaccine was received and its convenience and whether someone known to participant accompanied them; and willingness to receive the vaccine (if vaccine has not been received) and, if not, the reason why not (new items).

***11. Final questions***

Open-ended questions will seek insights on the general impact of the pandemic on the individual’s life and about their most anticipated activity following the end of the pandemic (new items).

### Procedure

Building on the first IDS-TILDA COVID-19 survey and previous waves of the longitudinal study, field researchers will be trained to administer the survey sensitively and consistently. Following information on the background to IDS-TILDA and the COVID-19 survey, they will be instructed on their roles and responsibilities. They will be given training in the use of the survey software. Training on re-affirming consent, maintaining confidentiality and data protection will be provided, as well as instruction on what to do if there are serious concerns about a participant’s mental health. The field researchers will be given the opportunity to roleplay the survey to practice its administration.

All participants will be contacted and their participation requested. Participants will either complete the survey by themselves, with help from someone who can assist them in answering the questions, or a proxy will respond on their behalf where the participant is unable to respond to the questions themselves. Consent will be reaffirmed verbally with participants before the survey begins. Written consent/assent was previously obtained for all participants to take part in Wave 4 of IDS-TILDA. A system of process consent will be in place, whereby the field researchers will check at regular intervals during the interview whether the participant is happy to continue participation, and will be alert to signs that the participant may not wish to proceed with further questions. The participants will be reminded at the outset that they are not obliged to answer any specific questions they do not wish to, and can withdraw from the interview at any point. A consent declaration was obtained from the Health Research Consent Declaration Committee to facilitate the inclusion of individuals with intellectual disability. Easy read materials will be developed to assist participants in responding to the questions. Pilot testing with a research assistant with intellectual disability has indicated that the survey should take approximately 45–60 minutes to complete. The survey will be conducted via telephone/video call, and interviewers will enter the survey data into a software system.

### Data analysis and statistical plan

Descriptive statistics will be calculated to describe the effects of the COVID-19 pandemic on the IDS-TILDA participants. Significance testing will help determine the level of differences between the first and second questionnaires where items are in common.

### Dissemination plan

A report will be prepared on the results of this survey, similar to that which described the results of the previous survey ( McCarron *et al.*, 2020). Updates will be provided to relevant stakeholders including the Department of Health. Results of this survey will also be published in academic journals, presented at academic conferences, and included in a webinar presentation on COVID-19 and people with ID offered by the authors.

## Discussion

Among the key findings of our first survey, conducted from May-September 2020, were that most participants had been tested for COVID-19 but very few tested positive, there were no reports of participant mortality due to COVID-19, and a slight majority of participants reported positive aspects of the lockdown, but a similar proportion reported feeling stress/anxiety. The results of this second survey are likely to enhance our understanding of the impact over time of the COVID-19 pandemic and associated lockdowns on people ageing with an intellectual disability in Ireland. The survey will enhance our knowledge of infection control behaviours, the impact of the lockdown on social interaction and loneliness, and risk factors such as frailty and stressful life events. This survey will probe in greater depth about physical and mental health, as well as the level and nature of healthcare utilisation during the pandemic. The findings of this second questionnaire will be further enriched by information gathered on vaccination, including rates of vaccination, which vaccines are being employed, and what it means for people ageing with an intellectual disability to have received the vaccine. There are already similar research efforts underway in other countries and it will be important to compare findings as well as identify issues that have been unique to Ireland.

Notwithstanding the progression of vaccination, there may also be long-lasting effects of the pandemic and associated lockdowns, whether through “long COVID” or the psychosocial consequences of long periods of isolation from family or friends and the disruption of daily life including day services and social activities. The findings here will provide a first picture of such experiences of people with intellectual disability in Ireland, and will inform rehabilitative models to support people post-lockdown, as well as informing the rebuilding of services (e.g. can an increasing engagement with communication technology be used to enhance healthcare provision and reduce loneliness). The first survey already provided an evidence base for the prioritisation of vaccinations for people with an intellectual disability in Ireland. Although some lockdown measures are currently being lifted in Ireland, the possibility that more restrictive rules may later be introduced (e.g. due to new strains of the coronavirus) cannot be ruled out, and so planning for future lockdowns is required. At time of writing, planning is underway for Wave 5 IDS-TILDA, which will include an opportunity to gather more detailed information on the health and well-being of participants who experienced the pandemic and to further assess the long-term consequences of the pandemic.

## Study status

Data collection is currently underway for this study. We anticipate that data collection will be complete by the end of August 2021.

## Data availability

No participant data are associated with this article.
