# Supplementary material for: The impact of COVID-19 on people ageing with an intellectual disability in Ireland: Protocol for a follow-up survey
Source: HRB Open Res. 2021 Oct 15;4:95. Originally published 2021 Aug 26. [Version 2] doi: 10.12688/hrbopenres.13340.2 (PMC8456373; doi:10.12688/hrbopenres.13340.2)
Supplement: Peer review history

# Peer review: Reviewer response for version 2

**DOI:** 10.21956/hrbopenres.14644.r30629

**Published:** 2021-10-18

**Authors:** Angelo Carfi

**Affiliations:** 1 Centro Medicina Dell'Invecchiamento, Fondazione Policlinico Universitario Agostino Gemelli IRCCS, Università Cattolica del Sacro Cuore, Rome, Italy

Nothing to add.

Is the study design appropriate for the research question?

Yes

Is the rationale for, and objectives of, the study clearly described?

Yes

Are sufficient details of the methods provided to allow replication by others?

Yes

Are the datasets clearly presented in a useable and accessible format?

Not applicable

Reviewer Expertise:

Medical care of adults with Down syndrome and other intellectual disabilities

I confirm that I have read this submission and believe that I have an appropriate level of expertise to confirm that it is of an acceptable scientific standard.

# Peer review: Reviewer response for version 1

**DOI:** 10.21956/hrbopenres.14528.r30146

**Published:** 2021-09-21

**Authors:** Yona Lunsky

**Affiliations:** 1 Azrieli Adult Neurodevelopmental Centre, Centre for Addiction and Mental Health, Toronto, ON, Canada

This is a very timely protocol, building off a unique cohort study of older adults with intellectual disabilities. The authors provide a strong justification and overview for their study, which is essentially a more in depth follow-up of the pandemic experience of a well studied group of adults in Ireland. They select several key topics: vaccination experience, Long COVID symptoms, and physical and mental health of aging adults. Each of these are important, and not well covered by the studies focused on the earlier stages of the pandemic. Furthermore, other studies tend to focus on younger adults and children, and few studies include the perspective of adults with ID, based on such a large cohort. 

 I particularly appreciated the inclusion of an advisory to select the measures and topics covered, and the various steps to ensure informed consent including "process consent." Some clarification on what the Health Research Consent Declaration Committee does would be helpful.

 It would also be helpful to include a little bit more information about how health and social services are structured in Ireland and more detail on the IDS TILDA cohort. To contextualize findings, it would be good to know the types of congregate care there, and the proportion of people in the cohort who live in such settings. Low rates of COVID-19 in the cohort may be a reflection of overall rates in Ireland, or on the level of care and support available to people in the IDS-TILDA study relative to other adults with ID. Representativeness of the sample would be important to discuss in any reports based on this protocol.

 With regard to measurement, it would be good to describe which measures designed for the general population have been adapted for people with ID (e.g., EPII, GAD-7, UCLA Loneliness scale). It would also be helpful to explain how analyses of self reported experiences will be conducted separately from when an informant responds to the items on behalf of someone with a disability. 

 There will be important lessons to learn from results of this wave of the IDS-TILDA study, relevant to other jurisdictions. I look forward to reading results when they are available.

Is the study design appropriate for the research question?

Yes

Is the rationale for, and objectives of, the study clearly described?

Yes

Are sufficient details of the methods provided to allow replication by others?

Yes

Are the datasets clearly presented in a useable and accessible format?

Yes

Reviewer Expertise:

Intellectual disability, health

I confirm that I have read this submission and believe that I have an appropriate level of expertise to confirm that it is of an acceptable scientific standard.

# Response

**Published:** 2021-10-07

**Authors:** Andrew Allen

**Affiliations:** Trinity College Dublin, Ireland

We thank this reviewer for their positive appraisal of the article and for their approval.

 This reviewer has noted a number of possible additions/changes that could be benefit the protocol paper, and we believe these have indeed improved the quality of this protocol. We quote the comments and outline our responses below.

*“Some clarification on what the Health Research Consent Declaration Committee does would be helpful.”*

 We have added information on the role of the Health Research Consent Declaration Committee and the meaning of a consent declaration from this body, with a link to their website (see Procedure, second paragraph).

*“It would also be helpful to include a little bit more information about how health and social services are structured in Ireland and more detail on the IDS TILDA cohort. To contextualize findings, it would be good to know the types of congregate care there, and the proportion of people in the cohort who live in such settings. Low rates of COVID-19 in the cohort may be a reflection of overall rates in Ireland, or on the level of care and support available to people in the IDS-TILDA study relative to other adults with ID. Representativeness of the sample would be important to discuss in any reports based on this protocol.”*

 This is a valid point about settings in which people live. We have added the information to the introduction, fourth paragraph. As noted on pages 4 and 5, the IDS-TILDA sample as a whole is representative of people with intellectual disability aged 40 or over in Ireland.

*“With regard to measurement, it would be good to describe which measures designed for the general population have been adapted for people with ID (e.g., EPII, GAD-7, UCLA Loneliness scale).”*

 Measurements or questions designed for the general population were not directly edited for accessibility. However, easy read materials for the overall questionnaire were developed, in consultation with the Inclusive Research Network, to assist people with intellectual disability in completing the survey (see Procedure, second paragraph). It is also the IDS-TILDA practice to pilot instruments and easy read materials with individuals with intellectual disability to ensure these accessibility steps are sufficient.

*“It would also be helpful to explain how analyses of self reported experiences will be conducted separately from when an informant responds to the items on behalf of someone with a disability. “*

 We will look at overall trends in the data from the whole sample; we will also compare self-reported to proxy responses for key variables (e.g. anxiety or depression), similar our analysis of the data from the first phase (McCarron *et al.,* 2020).

# Peer review: Reviewer response for version 1

**DOI:** 10.21956/hrbopenres.14528.r30144

**Published:** 2021-09-13

**Authors:** Angelo Carfi

**Affiliations:** 1 Centro Medicina Dell'Invecchiamento, Fondazione Policlinico Universitario Agostino Gemelli IRCCS, Università Cattolica del Sacro Cuore, Rome, Italy

The manuscript outlines the methodology by which the second wave of a survey dedicated to assessing the impact of the Covid-19 pandemic on adults with intellectual disabilities will be conducted. The topic is of great interest as the impact of the pandemic on this often overlooked segment of the population has been extremely heavy.

 The proposed methodology has many strengths:

- It is based on the experience of a very long running longitudinal study called IDS-TILDA.
- It has a longitudinal design.
- It has a holistic approach to the person with ID and investigates multiple domains impacted by the pandemic.
- I believe this methodological approach can serve as a useful example and incentive for other similar studies, and I anticipate that the data from this research will be extremely informative.

Is the study design appropriate for the research question?

Yes

Is the rationale for, and objectives of, the study clearly described?

Yes

Are sufficient details of the methods provided to allow replication by others?

Yes

Are the datasets clearly presented in a useable and accessible format?

Not applicable

Reviewer Expertise:

Medical care of adults with Down syndrome and other intellectual disabilities

I confirm that I have read this submission and believe that I have an appropriate level of expertise to confirm that it is of an acceptable scientific standard.

# Response

**Published:** 2021-10-07

**Authors:** Andrew Allen

**Affiliations:** Trinity College Dublin, Ireland

We thank this reviewer for their positive appraisal of the article and for their approval.

 As there are no issues to be addressed reported by this author, we have not made any corresponding changes to this study protocol.
